# Generation of ultra-sound during tape peeling

**DOI:** 10.1038/srep04326

**Published:** 2014-03-21

**Authors:** Jeremy O. Marston, Paul W. Riker, Sigurdur T. Thoroddsen

**Affiliations:** 1 Division of Physical Sciences and Engineering, King Abdullah University of Science and Technology, Thuwal 23955-6900, Kingdom of Saudi Arabia; 2 Visualization Core Lab, King Abdullah University of Science and Technology, Thuwal 23955-6900, Kingdom of Saudi Arabia

## Abstract

We investigate the generation of the screeching sound commonly heard during tape peeling using synchronised high-speed video and audio acquisition. We determine the peak frequencies in the audio spectrum and, in addition to a peak frequency at the upper end of the audible range (around 20 kHz), we find an unexpected strong sound with a high-frequency far above the audible range, typically around 50 kHz. Using the corresponding video data, the origins of the key frequencies are confirmed as being due to the substructure “fracture” bands, which we herein observe in both high-speed continuous peeling motions and in the slip phases for stick-slip peeling motions.

Tape peeling is a simple yet beautiful example of the fracture process, a very complex phenomena which can be viewed across many different scales^1^. Moreover, the peeling of an adhesive tape is now known to exhibit some truly unexpected physics, such as the emission of X-rays^2^.

In general, two different types of peeling motion can be observed - namely – “stick-slip” or “continuous” peeling. The former has been the subject of significant study^1^,^3^,^4^,^5^ for typically low peeling speeds of *O*(10) cm/s due to the production of a characteristic sound audible to the human ear, whilst the latter has been less studied. For extremely low peeling speeds (*O*(10^−5^) m/s), a sawtooth pattern was observed at the peeling front^6^.

Acoustic sampling of tape peeling^1^ allowed extraction of the periodicity (i.e. frequency) of the stick-slip cycles for various velocities, whilst direct high-speed imaging (at 16 kfps) of the stick-slip motion was performed^5^ to extract the period and duration of the slip and stick phases, allowing construction of spatiotemporal representations of the peeling point. Further to these studies, ultra-high-speed imaging at up to 1 Mfps was used^7^ to reveal new insight into the stick-slip motion, with the observation of intermediate or “sub-structure” fracture bands which occur in the slip phase and move rapidly across the width of the tape at speeds up to 500 m/s.

We now extend these previous experimental studies by performing concurrent (synchronised) high-speed video imaging and audio acquistion of rapid tape peeling where the typical peeling speeds are between 3 and 15 m/s. We examine both peeling regimes (stick-slip and continuous), with an emphasis on determining the origins of the peak acoustic frequencies. Surprisingly, we find the emergence of an unexpectedly strong sound in the ultrasonic domain with typical frequencies around 50 kHz.

## Experimental details

### Basic setup

**Figure 1:**
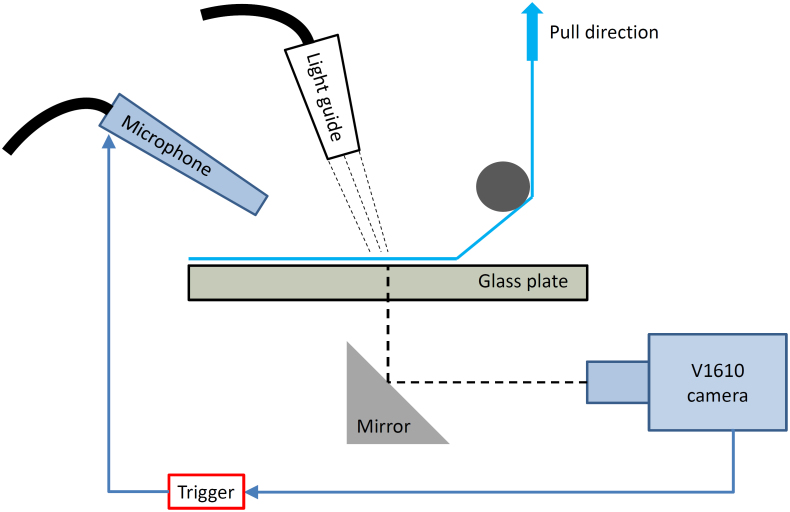
Schematic representation of the experimental setup used. The tape, shown in blue was attached to the top of a thick transparent glass plate. The microphone was placed approximately 15 cm from the viewing region.

We use an experimental setup, shown in Figure 1, similar to that used in^7^, whereby a strip of tape was initially stuck onto a thick, transparent glass plate which in turn was clamped into a solid steel mount approximately 10 cm above an optical table. This allowed placement of a 45*°* mirror underneath for optical access. Illumination from above was diffused by the tape itself, rendering good contrast in the video sequences. The tape was then peeled rapidly by pulling upward on the free end, whilst a bar placed 3–5 cm above and away from the viewing region on the glass plate ensured a reproducible angle (30–45*°*) between the glass plate and the detached tape for a given number of repeat trials. Four different tapes were used herein - namely - 3M Scotch Invisible tape, 3M Scotch transparent, Sellotape Invisible and HomeLife Invisible. All tapes have similar backing and adhesive layer thicknesses (~20 *μ*m).

### Video

We capture the peeling events using a high-speed video camera (Phantom V1610) operating at 48,000 fps. This frame rate was chosen due to the requirement of an exact number of audio samples per video frame, whereby the audio sampling rate was 192 kHz. Both the video and audio recordings were started by a common trigger using the camera's image-based auto-trigger (IBAT). The selection of a Nikon Micro-Nikkor 105 mm lens rendered an effective pixel resolution of 30 *μ*m/px, yielding a total field-of-view of 1.2 × 1.85 cm.

### Audio

The sound generated by the peeling process was captured using a class I measurement microphone and an analog-to-digital converter with a sample rate of 192 kHz, allowing for the capture of frequencies up to 96 kHz. The microphone itself was placed 15 cm away from the location of the tape peeling in the view of the camera and the signal was captured using a Pro Tools digital audio workstation. The raw waveform signal was then analysed using ProTools and a custom-built spectrograph application. The frequency spectrums and spectrograms were generated by performing a fast Fourier transform (FFT) and a wavelet transform, respectively, in order to identify the dominant frequencies for either the entire sequence (in the case of continuous peeling) or for individual slip events (in the case of stick-slip peeling). The audio samples were offset from the actual trigger point to account for the speed of sound in air due to the location of the microphone and we verified that this offset was constant for a number of different realisations.

## Observations from video data

**Figure 2:**
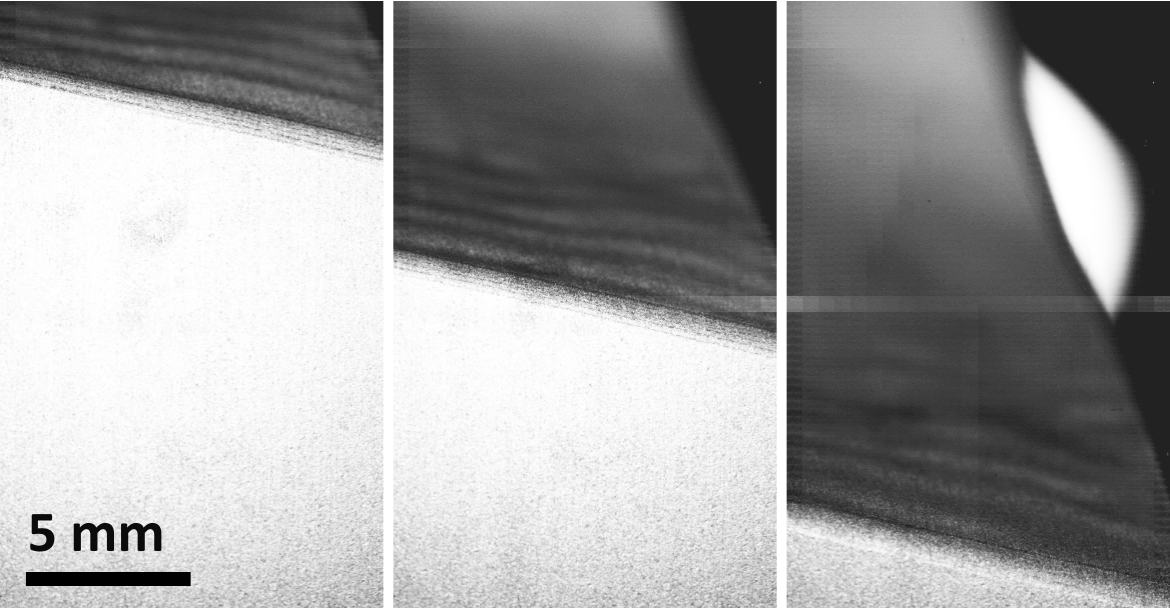
Frames taken from a video sequence recorded at 48,000 fps. The time between successive frames here is 2 ms. Each frame corresponds to the beginning of a slip event.

**Figure 3:**
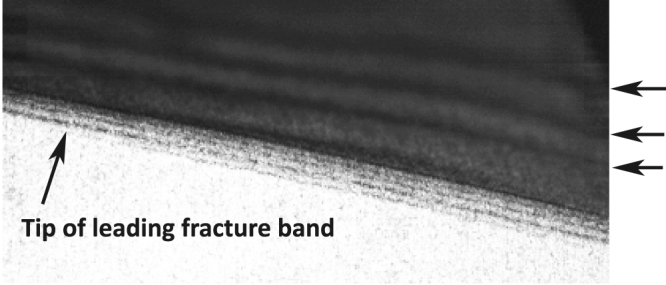
Close-up view of the detachment front from the same sequence as Figure 2. The fracture bands propagate from right to left. The waves in the detached tape are clearly visible, indicated by the arrows to the right of the image.

Select frames from a typical video sequence are shown in Figure 2. Here, the direction of the detachment front is from top to bottom (see also the supplemental movie for this Figure). Each of these frames shows the initial motion of detachment at the beginning of discrete “stick-slip” regions, where we observe the substructure or “fracture” bands, first reported in^7^. Figure 3 shows a close-up view of this phenomenon from a frame taken 40 *μ*s prior to the sequence shown in Figure 2. The fracture bands are clearly visible and stream from right-to-left in this realisation. It is precisely these bands that result in waves in the detached tape, which have been indicated by the arrows on the right.

Whilst we cannot observe the motion of the tip of the fracture bands frame-by-frame, due to insufficient temporal resolution, we estimate their speed by noting that each band travels the entire width of the video frame within one frame, thus giving speeds of up to 500 m/s, in agreement with previous calculations^7^. In all experimental trials, whether the peeling regime is stick-slip *or* continuous, we find that the fracture bands are equally spaced apart by approximately *L_fb_* = 120 − 200 *μ*m, depending on the brand of tape being used but that it is constant for each brand. The exact cause of this particular spacing is currently unknown, however, it is expected to be intimately linked to the adhesive and rheological properties of the tape since it appears to be more or less independent of the peeling speed and thus the applied load.

**Figure 4:**
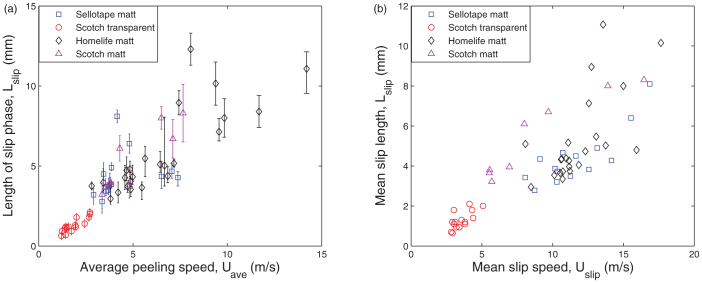
Length of slip phases plotted against (a) average peeling speed and (b) slip speed for the discrete slip events. The data here incorporates four different tapes.

In general, there is monotonic increase in the length of the slip phase with peeling speed or equivalently, with the speed of the slip phase, as shown in Figures 4 (a) and (b). This is to be expected as one would expect to see longer slip phases with increasing speed as the peeling motion transitions towards the continuous peeling regime. Within each of these slip phases, there is an exact number of fracture bands that occur at a consistent separation distance, *L_fb_*, and it is the speed of each slip phase and this separation distance which determines the frequency of the sound, as we show herein.

## Audio data

**Figure 5:**
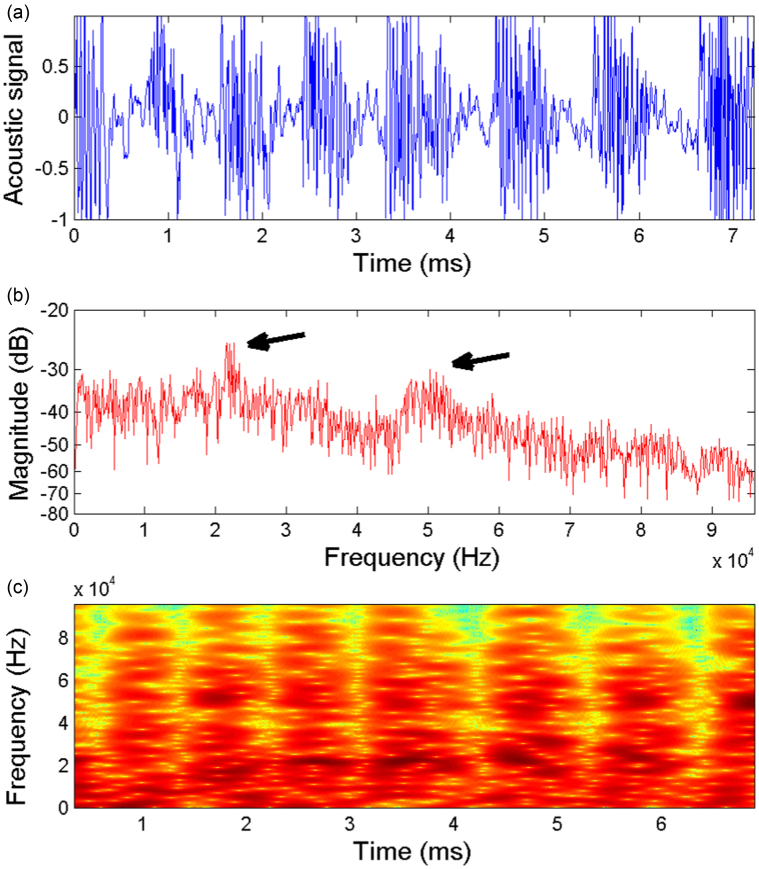
Raw waveform (acoustic signal) data for the same realisation as Figure 2, covering the entire length of video sequence. The individual stick-slip events are clearly detectable in the raw signal, whilst in the frequency spectrum (b) and spectrograph (c), we can identify two distinct peak frequencies at approximately 22 kHz and 50 kHz as indicated by the black arrows in (b) and the dark regions in (c). The tape was 3M Scotch Invisible tape.

A plot of the raw time-domain acoustic signal from the same realisation as in Figure 2 is shown in Figure 5(a) and the corresponding frequency spectrum and frquency-time spectrograph in Figures 5(b) and 5(c), respectively. This data (1388 audio samples) encompasses the entire length of the captured video sequence (7.2 ms) and clearly depicts a series of discrete events, which are precisely the “slip” phases observed in the video. This is verified by checking the exact start time and duration of each of the slip events in the video and audio data, which coincided exactly after taking into account the speed of sound offset due to the location of the microphone. The frequency spectrum clearly shows a first peak frequency at 22 kHz, which is also observed as the dark regions in the 3rd, 4th, 5th and 6th slip phases in Figure 5(c).

**Figure 6:**
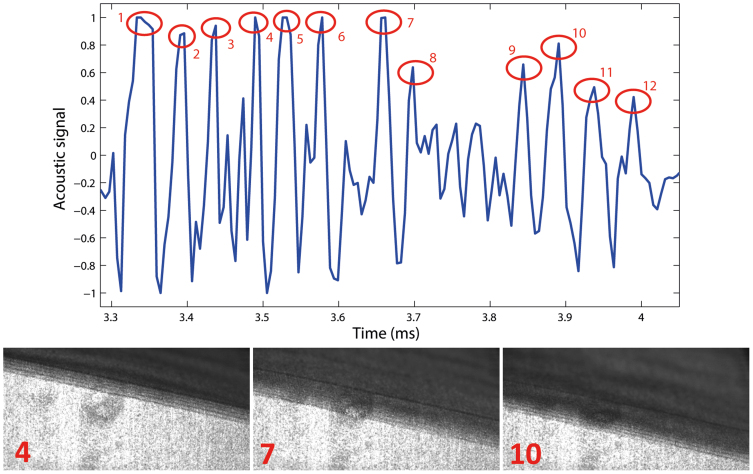
Raw audio signal for the 4th individual slip event, from the same realisation as Figure 5(a) with corresponding images showing completed fracture bands 4, 7 and 10 out of a total of 12 for this particular slip cycle. There is a one-to-one correspondance between the number of peaks in the audio signal and the number of fracture bands observed in the video sequence for this slip cycle. The markers on this plot correpsond to the generation of the peak audio frequency around 20 kHz.

In order to examine the origins of this frequency in more detail, Figure 6 shows the raw audio signal from the 4th individual slip cycle, where we can clearly make out 12 discrete peaks, which corresponds precisely to the number of fracture bands that occur during this slip cycle. The images below show the progression of the peeling front with the number of completed fracture bands indicated in red. For this particular slip cycle we thus have 12 bands occurring in ~540 *μ*s, leading to a frequency of 22 kHz, which is precisely the peak seen in Figure 5(b) and dense region in Figure 5(c). Note that this frequency can also be calculated from the wavespeed and wavelengths in the detached tape, but since these are not always in focus in the video sequences and the fact that they are caused by the fracture bands anyway, we use the observations of the fracture bands as a more reliable method of predicting the audio frequency.

**Figure 7:**
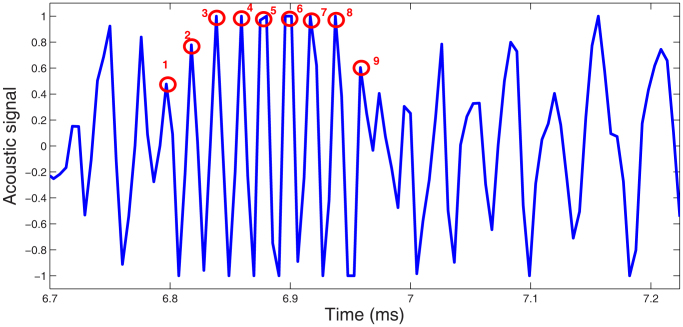
Raw audio signal for the final slip cycle captured in the same realisation as Figure 5(a). The markers on the peaks in this plot correspond to the generation of the peak audio frequency around 52 kHz.

Interestingly though, we also note another dominant high-frequency around 50 kHz, which occurs at approximately the same magnitude as the low-frequency (≤20 kHz) sound in the audible range. The spectrograph in Figure 5(c) further shows that this high-frequency sound, around 50 kHz, is most prominent in the 2nd, 6th and 7th discrete slip events for this particular trial. Inspection of the video sequences for these particular individual slip events indicates that the fracture bands occur at rates of 48–53 kHz, which is confirmed by inspection of the audio signal of these slip cycles, for example shown in Figure 7 for the 7th slip cycle. Here, the markers on the peaks in the middle of the cycle clearly correspond to the peak around 52 kHz in Figure 5(b).

**Figure 8:**
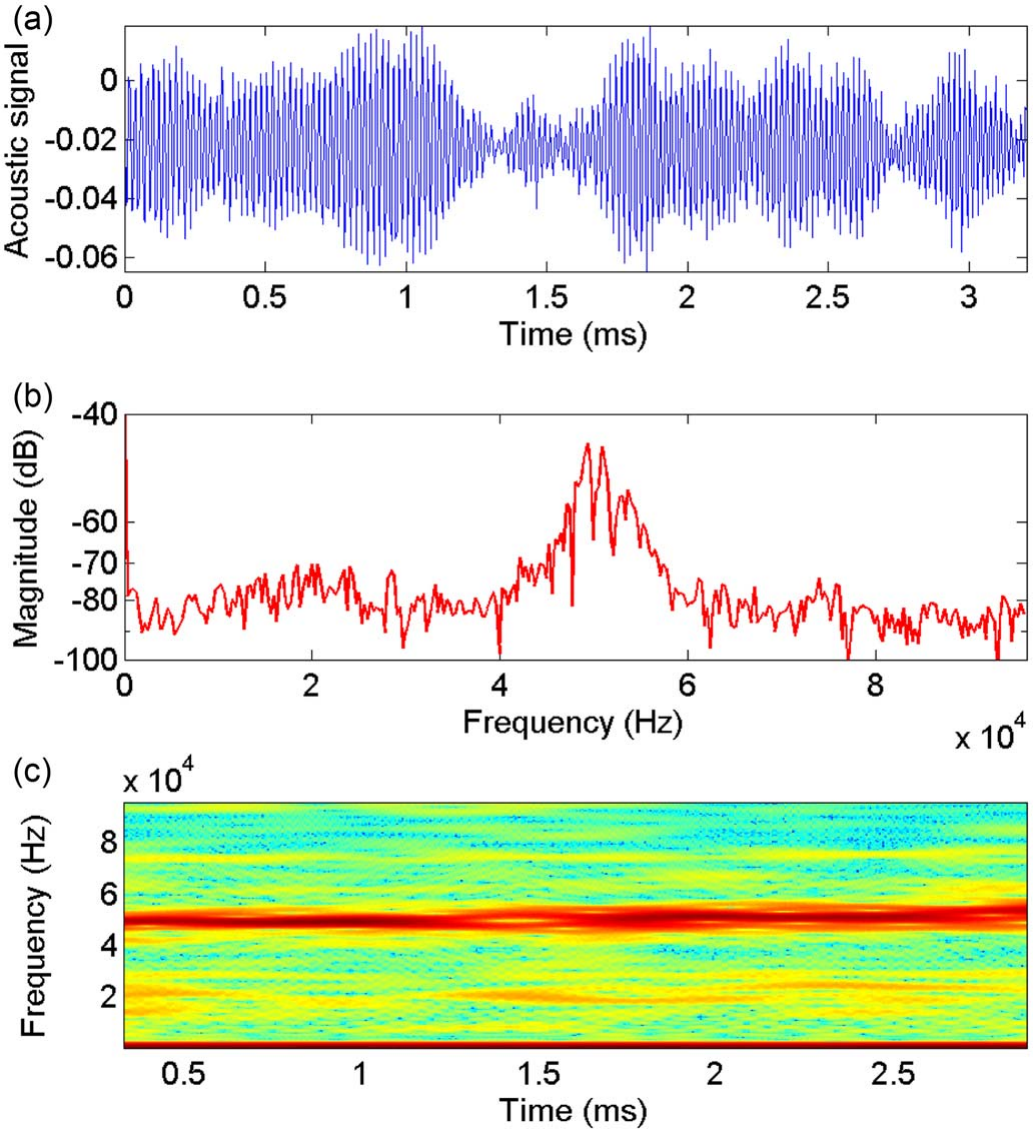
Raw signal, frequency spectrum and frequency-time spectrograph for a continuous peeling trial. For this realisation, *U_peel_* = 7.2 m/s. The tape was 3M Scotch transparent tape.

In contrast to this stick-slip motion, the continuous peeling regime exhibits a qualitatively different frequency spectrum, as shown by Figure 8, which shows the equivalent raw signal, frequency spectrum and spectrograph for a continuous peeling trial. Here, we can see a very clear peak in the frequency spectrum and spectrograph centred around 49 kHz. In this particular realisation, the peeling speed *U_peel_* = 7.2 m/s and the spacing between the individual fracture bands *L_fb_* ≈ 150 *μ*m, thus giving a rate of generation of the bands of *U_peel_*/*L_fb_* = 48 kHz, again in excellent agreement with the captured peak audio frequency.

**Figure 9:**
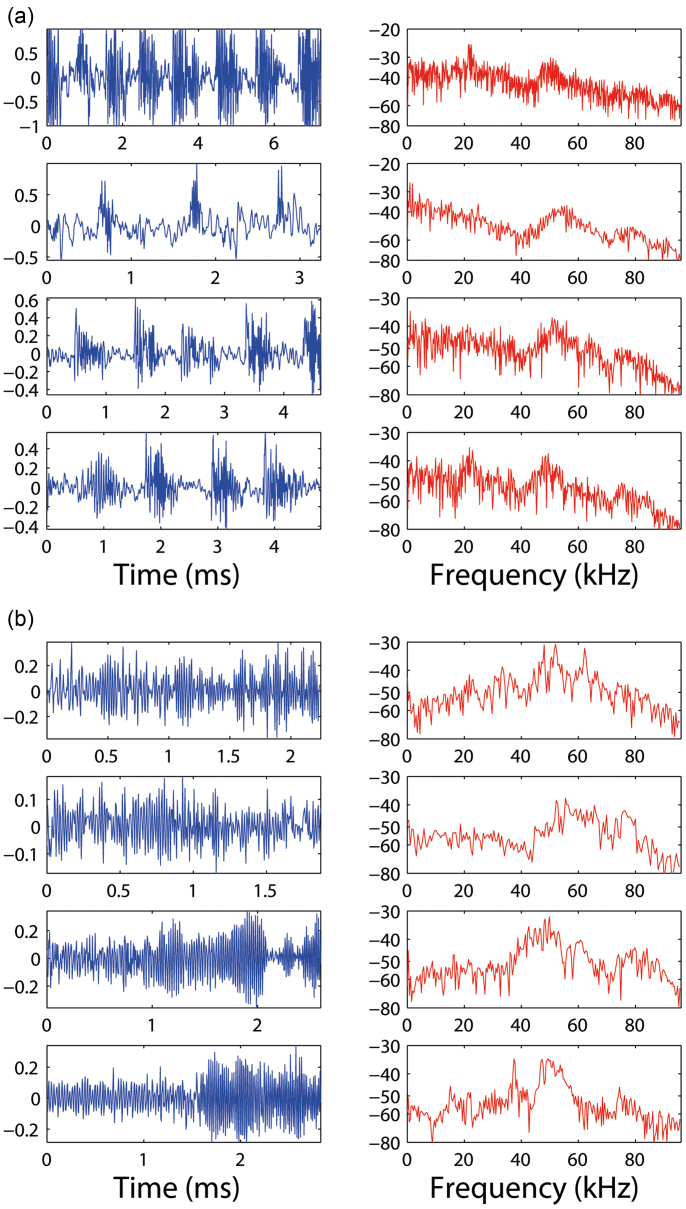
Four different realisations of (a) stick-slip peeling and (b) continuous peeling motion with both raw waveforms (left) and frequency spectrums (right), all showing high-strength signals in the ultrasonic region. All trials were performed with 3M Scotch Invisible tape.

Figures 5 and 8 clearly show a dramatic difference between the two peeling regimes, which is a consistent feature across all brands of tapes and highlighted further in Figures 9 (a) and (b) showing both raw acoustic signals and corresponding frequency spectra for four realisations from both stick-slip (Figure 9(a)) and continuous peeling (Figure 9(b)) motions. The raw signals clearly represent the difference between the two types of motion, whereby the periodic structure seen in the stick-slip peeling is absent for the continuous peeling examples. Moreover, the frequency spectra for the continuous peeling examples exhibit much more pronounced peaks in the high-frequency regime, between 40 and 60 kHz, compared to stick-slip and it is noted that the strength of this sound is actually higher than the audible-range frequencies.

**Figure 10:**
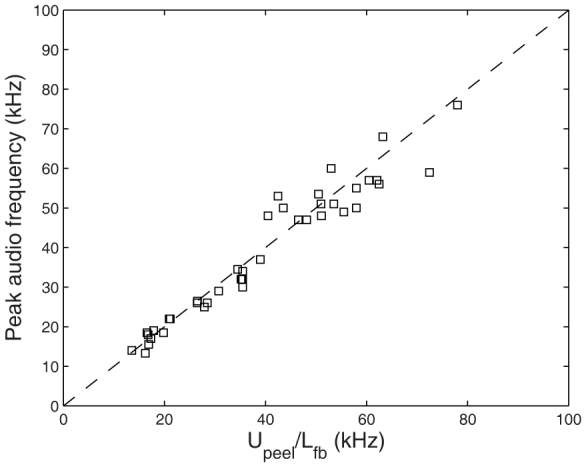
Peak frequency from the acoustic signal versus the generation rate of the fracture bands, *U_peel_*/*L_fb_*, for the continuous peeling regime. The dashed line indicates parity.

As with Figure 8, we can rationalize this observation by using the video data to assess the average speed of the peeling front in the continuous peeling regime, whereby *U_peel_* ≈ 10 m/s. Recalling that the spacing of the individual fracture bands is *L_fb_* ≈ 200 *μ*m, we see that the generation of the fracture bands occurs at a rate of approximately 50 kHz, thus corresponding very closely to the peak frequency of the captured acoustic signal for these trials. As such, we confirm the presence of both audible and ultrasonic sound, where the peak frequencies can both be attributed to the fracture bands which occur both in continuous peeling motions *and* in the stick-slip peeling during the individual slip events. This is shown graphically in Figure 10, where we have plotted the peak captured frequency for multiple tapes against the predicted frequency based upon the generation rate of the fracture bands, *U_peel_*/*L_fb_*.

Finally, by using a cross-over filter (a pair of symmetrical low-pass and high-pass 3rd order filters) at 20 kHz, we estimate the total percentage of the sound energy in the ultrasonic domain to be between 13–40% for stick-slip trials, and between 46–79% in most cases for continuous peeling, showing that irrespective of the peeling motion, there is a significant portion of sound energy above the human audible range.

## Conclusions

In summary, we have performed experiments to extend previous revelations about the peeling of an adhesive tape. Both continuous peeling and stick-slip regimes were observed and we have confirmed the presence of the fracture bands first observed by Thoroddsen et al.^7^ in both. Furthermore, audio acquisition concurrent with imaging allowed us to assess the sound generated in this process and, in line with the conjecture in^7^, there is sound in the ultrasonic domain. Moreover, and quite surprisingly, this sound is as strong and, in many cases, stronger than that in the audible domain. By cross-examining the audio signals with the video data, we find that the substructure fracture bands are responsible for the dominant frequencies in the sound generated in this process. This finding adds to the unexpected and fascinating physics of this seemingly simple and ubiquitous phenomena. Correlating the peeling speed with the peak audio frequencies for a range of substrates and applied loads is the subject of ongoing work and will be addressed in the future.

## Author Contributions

S.T.T. concepted the research, J.O.M. and P.W.R. carried out the experimental work and analysis. J.O.M. prepared the figures and wrote the manuscript. All authors reviewed the manuscript.

## Supplementary Material

Supplementary Information

Supplementary Information
