# Supplementary material for: Generation of ultra-sound during tape peeling
Source: Sci Rep. 2014 Mar 21;4:4326. doi: 10.1038/srep04326 (PMC3961747; doi:10.1038/srep04326)
Supplement: Supplementary Information [file srep04326-s1.docx]

**Generation of ultra-sound during tape peeling**

Jeremy O. Marston^1^, Paul W. Riker^2^ & Sigurdur T. Thoroddsen^1^

^1^Division of Physical Sciences and Engineering, King Abdullah University of Science and Technology, Thuwal 23955-6900, Kingdom of Saudi Arabia

^2^Visualization Core Lab, King Abdullah University of Science and Technology, Thuwal 23955-6900, Kingdom of Saudi Arabia

Figure 2 supplemental video caption:

Video sequence showing the stick-slip peeling motion of Scotch Tape, recorded at 48000 fps. The field of view is 1.2 x 1.85 cm. A total of 5 slip phases can be seen, each exhibiting the sub-structure fracture bands. See also Figures 2 and 3 in the main text of the manuscript.
